# Rising clindamycin resistance in group A Streptococcus in an Irish healthcare institution

**DOI:** 10.1099/acmi.0.000772.v4

**Published:** 2024-06-27

**Authors:** Susan Lapthorne, Robert McWade, Nuala Scanlon, Saoirse Ní Bhaoill, Aoife Page, Clare O'Donnell, Gabriela Dornikova, Margaret Hannan, Breda Lynch, Maureen Lynch, Deirdre Brady

**Affiliations:** 1 Clinical Microbiology Department, Mater Misericordiae University Hospital, Dublin 7, Ireland; 2 Pharmacy Department, Mater Misericordiae University Hospital, Dublin 7, Ireland; 3 Associate Clinical Professor, UCD School of Medicine, University College Dublin, Dublin, Ireland

**Keywords:** clindamycin, group A Streptococcus, *Streptococcus pyogenes*: antimicrobial resistance

## Abstract

Group A streptococcus (GAS) can cause serious invasive disease in humans with a high mortality rate. An increase in GAS infections was reported in Ireland in 2022, and this increase has been sustained in 2023 and is paralleled by similar trends in Europe. Rising antimicrobial resistance is a global problem and presents significant challenges to clinicians treating GAS infection. There was a reported increase in clindamycin resistance in GAS isolates in Ireland in 2022. We examined antimicrobial susceptibility patterns of GAS isolates in our institution in 2022. Although all GAS isolates included in our study were susceptible to penicillin, we noted a high clindamycin resistance rate of 28 % in our invasive GAS isolates. We also noted high tetracycline and erythromycin resistance, 43 and 30 %, respectively. Our results could have implications for empiric antimicrobial prescribing guidelines for skin and soft tissue infections, which often include clindamycin as it inhibits the production of many virulence factors associated with GAS. In addition, macrolides are often the first line recommended antibiotic for patients with anaphylaxis to penicillin. This study emphasises the importance of continuous surveillance and antimicrobial susceptibility testing of invasive and non-invasive isolates in order to monitor trends in increasing antimicrobial resistance.

## Data Summary

The authors are of the opinion that all information required to understand the article is included in the article. For interest, consistent with Access Microbiology’s Open Data Policy, we have published the full list of susceptibility results for our GAS isolates on Figshare at the following DOI 10.6084/m9.figshare.25809451[1].

## Introduction

Group A Streptococcus (GAS), also known as *Streptococcus pyogenes*, is a facultative anaerobic Gram-positive coccus that is a common coloniser of the human throat and skin. It is commonly associated with pharyngitis and skin and soft tissue infections, which are not typically invasive. However, less commonly, GAS can cause invasive disease including life-threatening necrotising fasciitis and streptococcal toxic shock syndrome [2,3].

Since 2004, invasive GAS (iGAS) has been a notifiable disease in Ireland. Incidence rates ranged from 0.8 to 1.65 per 100  000 in 2004–2011 with a rise in cases from 2012 to 2019 to 2.7–3.7 per 100  000 [4,5]. In 2020–2021, there was a decrease in incidence to 0.9–0.71 per 100  000, likely as a result of COVID-19 containment measures [5]. In 2020 and 2021, there were 40 and 33 notified cases, respectively [6]. However, in 2022, there was a 3.7-fold increase in incidence, with 123 cases, the majority of which occurred from October to December of that year [6]. This upsurge was sustained in 2023, with 518 cases recorded which represents a 4.2-fold increase compared to 2022 [6]. This was echoed across Europe, with Northern Ireland, England, the Netherlands, Sweden, France and Spain also reporting an increased incidence of iGAS infections [7,9].

The Irish Meningitis and Sepsis Reference Laboratory (IMSRL) performs sequence typing, species confirmation and susceptibility testing on invasive and non-invasive isolates that are sent to the reference laboratory [5]. All invasive and non-invasive GAS isolates received in the reference laboratory from 2012 to 2021 were susceptible to penicillin. However, there was a notable increase in erythromycin resistance from 2–8 % in 2012–2019 to 15 % in 2020–2021. There was also an increase in clindamycin resistance from 2–5 % in 2012–2019 to 7.8 % in 2020–2021. This is significant because in iGAS infection, clindamycin is often given as an adjunct to penicillin as it inhibits the production of several GAS toxic virulence factors and thus has an antitoxin effect [10].

In our institution, we noted an increase in severe invasive GAS in 2022 in patients in the intensive care unit who were not responding clinically and biochemically to penicillin and clindamycin combination therapy. We therefore examined the susceptibility data in our GAS isolates from 2022 to determine if there was a change in the susceptibility pattern compared to the published national data and whether the empiric antimicrobial guidelines in our institution should be adjusted as a result of a change in susceptibility pattern.

## Methods

This was a retrospective study of all Group A Streptococcus (GAS) isolates identified and cultured from adult patients in our institution from January to December 2022. All general practice (GP), emergency department (ED), outpatient department and inpatient isolates were included in the study. Duplicate isolates from the same patient were excluded if they had the same susceptibility profile. The site of the isolate was recorded as well as any known clinical information. The Health Protection Surveillance Centre (HPSC) changed the definition of invasive GAS in December 2022 and so, for the purposes of this study, the 2021 HPSC case definition was used to determine if the isolates were from cases of invasive GAS [6].

GAS isolates were identified using matrix-assisted laser desorption ionisation (MALDI) time-of-flight mass spectrometry (MALDI-TOF MS; bioMérieux). Phenotypic antimicrobial susceptibility to penicillin, clindamycin, erythromycin, tetracycline and vancomycin was tested using the European Committee on Antimicrobial Susceptibility Testing (EUCAST) disc diffusion method [11]. Susceptibility results were interpreted according to EUCAST zone diameter breakpoints [12]. The susceptibility testing results for each isolate were collated and compared to the national published susceptibility data. Data were analysed using IBM SPSS Statistics (version 26), which was used to generate frequency tables and crosstabulations. This study was approved by the Mater Misericordiae University Hospital Clinical Audit and Effectiveness Committee.

## Results

During the year 2022, we cultured 121 distinct GAS isolates in our laboratory. General practice (GP) accounted for 49.6 % of isolates (*n*=60), the emergency department (ED) accounted for 33.9 % of isolates (*n*=41), whilst 11.6 % (*n*=14) of isolates originated from the outpatient department, and 4.9 % (*n*=6) originated from inpatients.

**Fig. 1.**
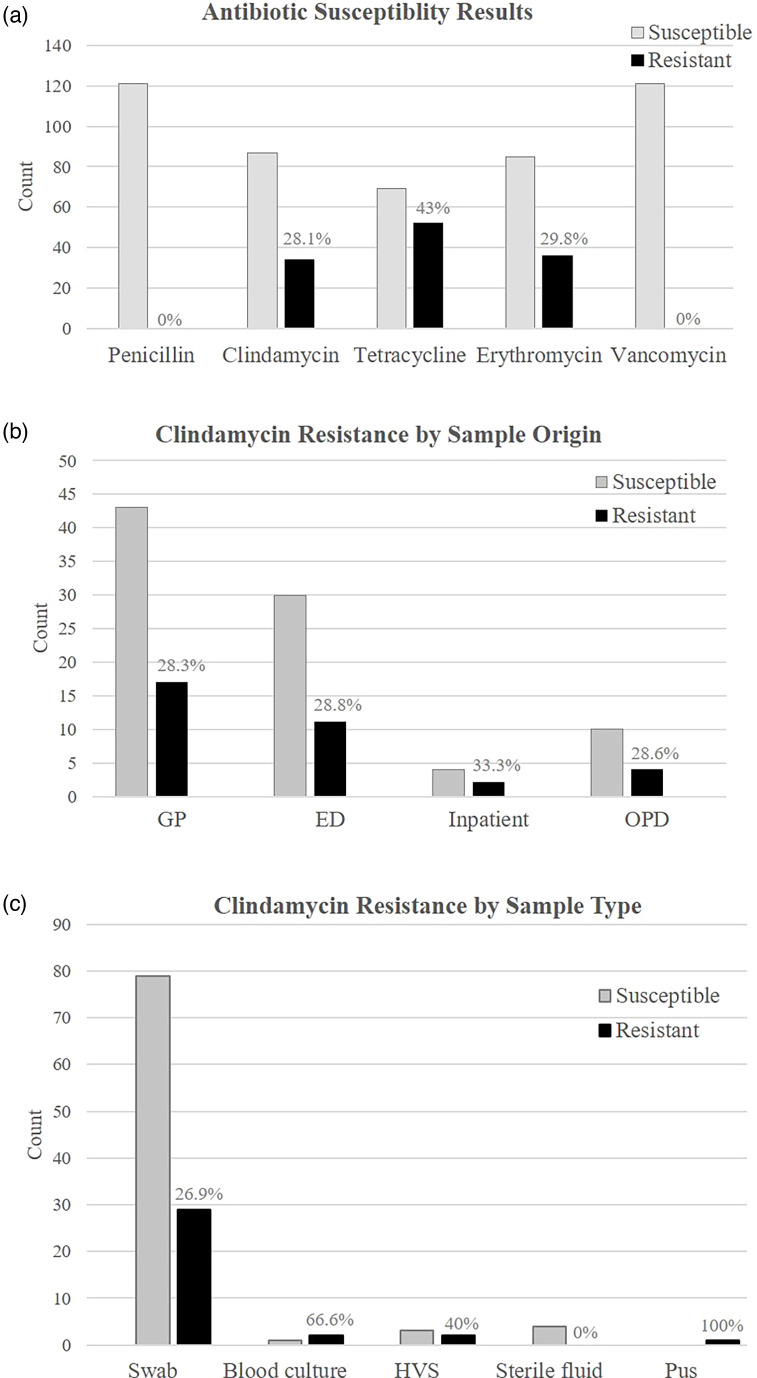
(**a**) Antibiotic susceptibility results for all isolates included in the study (*n*=121). (**b**) Clindamycin resistance rates by origin of the clinical isolate. (**c**) Clindamycin resistance rate by the sample type. Percentage resistance to each antibiotic is indicated above each resistance bar. GP, general practice; ED, emergency department; OPD, outpatient department; HVS, high vaginal swab.

In our study, 100 % (*n*=121) of invasive and non-invasive isolates were susceptible to penicillin and vancomycin (Fig. 1a). The overall rate of clindamycin resistance was 28.1 % (*n*=34), tetracycline resistance was 43 % (*n*=52), and erythromycin resistance was 29.8 % (*n*=36).

When clindamycin resistance data was examined in relation to the origin of the sample, we found a resistance rate of 28.3 % (*n*=17) from samples originating from GP, 28.8 % (*n*=11) from ED samples, 33.3 % (*n*=2) from inpatient samples, and 28.6 % (*n*=4) from outpatient department samples (Fig. 1b). We further examined clindamycin resistance in relation to the sample type (Fig. 1c). The majority of samples (89.3 %, *n*=108) were labelled as swabs, which were from throat/tonsils (*n*=67), arm (*n*=5), leg (*n*=11), ankle (*n*=3), foot (*n*=2), elbow (*n*=2), abscess (site not stated; *n*=3), chest (*n*=1), hand/finger (*n*=6), vulva (*n*=1), ear (*n*=2), head (*n*=2), lip/mouth (*n*=2) and unspecified (*n*=1). The clindamycin resistance rate was 26.9 % (*n*=29). High vaginal swabs accounted for 4.1 % (*n*=5) of the samples and the clindamycin resistance rate was 40 % (*n*=2). The remaining isolates were from blood cultures (2.5 %, *n*=3), which had a clindamycin resistance rate of 66.6 % (*n*=2), fluid from normally sterile sites (3.3 %, *n*=4), which had zero clindamycin resistance, and pus (0.8 %, *n*=1), which had 100 % (*n*=1) clindamycin resistance.

We also analysed our data for evidence of cross resistance. There were just 30 isolates (24.8 %) that were fully susceptible to clindamycin, tetracycline and erythromycin. There were 34 isolates (28 %) that were clindamycin and erythromycin co-resistant and 32 isolates (26.4 %) were tetracycline and erythromycin co-resistant. There was no isolate resistant to clindamycin but susceptible to erythromycin. Two of the invasive isolates, both of which were isolates from blood cultures, were resistant to clindamycin, erythromycin and tetracycline.

Based on the HPSC definition of a confirmed case of invasive GAS, there were seven isolates from normally sterile sites including blood, pleural fluid, joint aspirate and deep tissue obtained in the operating theatre that met the case definition for invasive GAS. The overall rate of clindamycin resistance amongst invasive isolates was 28.6 % (*n*=2), with both of the resistant isolates originating from blood cultures. *Emm* typing was performed in the IMSRL on the blood culture isolates (*n*=3) and these were identified as one isolate each of *emm*9, *emm*27, and *emm*87.

## Discussion

This study examined the antibiotic susceptibility of GAS isolates in 2022, when there had been an international increase in the incidence of iGAS infections. The IMSRL reported a clindamycin resistance rate of 7.8 % in 2020–2021 and the rate of resistance amongst our 2022 isolates was significantly higher at 28.1 %, albeit from a small number of clinical isolates. Given the potentially high mortality from invasive GAS, this has significant clinical implications in terms of empiric antibiotic choice if GAS is in the list of differential diagnoses or if susceptibility results for a known case of invasive GAS are pending.

The three most common *emm* types associated with the increased in incidence of invasive GAS in Ireland since 2022 have been identified as *emm*1, *emm*12, and *emm*28 [6], whereas our invasive blood culture isolates were identified as *emm*9, *emm*27, and *emm*87. Our *emm*9 and *emm*27 isolates were co-resistant to clindamycin, erythromycin and tetracycline. A study examining GAS isolates in Belgium demonstrated that *emm*9 exhibited an M and inducible (iMLS) macrolide resistant phenotype [13] and a South Korean study has shown that *emm*27 demonstrated atypical constitutive macrolide resistant phenotype (cMLS) [14]. The *emm*87 isolate in this study was fully susceptible to clindamycin, erythromycin and tetracycline which is consistent with the majority of *emm*87 strains included in a study from Spain where 88 of 89 isolates were fully susceptible to the three antimicrobials and just one exhibited erythromycin resistance [15]. Our study is limited by being a single centre study in an acute hospital where it is normal practice only to type invasive GAS isolates. We do not know whether our finding of *emm* types that differ to the three most common types in Ireland is due to sporadic uncommon cases or due to local outbreaks of these *emm* types in non-invasive disease with occasional invasive cases. As non-invasive isolates have not been typed, we cannot answer this question.

Clindamycin, a lincosamide antibiotic, is often used as adjunctive therapy to penicillin for its known antitoxin effect [10]. In addition, it is often recommended as the first-line single agent antimicrobial therapy for severe skin and soft tissue infections in a patient with a penicillin allergy. Linezolid, a synthetic oxazolidinone, also inhibits the production of many GAS virulence factors involved in systemic toxicity and tissue destruction and can be considered an alternative to clindamycin [16]. Prior to this study, linezolid susceptibility was not routinely tested in our laboratory unless specifically requested by the clinical microbiology or clinical team. Based on the findings of this study, we now test linezolid susceptibility if an isolate is found to be clindamycin resistant on initial susceptibility testing, allowing us to use linezolid as a second agent for severe iGAS infections.

In our institution, the empiric guidelines for severe skin and soft tissue infection in a patient with penicillin anaphylaxis recommended single agent clindamycin. As a result of this study, we have changed our empiric guidelines to combination clindamycin and vancomycin as we had zero vancomycin resistant isolates. In a patient with suspected necrotising fasciitis, the empiric guidelines for patients with penicillin anaphylaxis now recommend ciprofloxacin, clindamycin, linezolid and gentamicin until the target organism(s) and susceptibility results are known.

This study focused primarily on clindamycin resistance, however, we also observed an increase in tetracycline and erythromycin resistance, which are also used as alternatives to penicillin to treat GAS infections in patients with a penicillin allergy. We also found co-resistance to clindamycin, tetracycline and erythromycin in one-quarter of our isolates. The HSE community prescribing guidelines recommend use of clarithromycin in patients with pharyngitis or cellulitis with severe penicillin allergy [17,18]. In addition, in certain situations, following risk assessment, chemoprophylaxis may be offered to close contacts of cases of invasive GAS. For patients with reported penicillin allergy, macrolides are recommended [19]. With elevated resistance rates, there may be a delay to appropriate alternatives or failure of prophylaxis. Given that our rate of resistance for erythromycin was 29.8 %, we await with interest the IMSRL macrolide resistance rates for 2022 as this may have implications for community prescribing guidelines in cases of penicillin allergy.

A rise in GAS antimicrobials resistance poses a challenge to treatment strategies and may be due to several factors including selective pressure exerted by the widespread use of antibiotics in community and hospital settings. The increase in resistance that we observed in our institution may be a population-specific change and, therefore, notification data published by the HPSC and surveillance data generated and published by the IMSRL are crucial in monitoring the number of cases and dynamics of antimicrobial resistance nationally. An increase in clindamycin resistance in GAS underscores the importance of ongoing surveillance efforts to track resistance patterns, guide empirical treatment choices, and inform public health interventions. Furthermore, emphasis on antimicrobial stewardship programmes, aimed at optimising antibiotic use and minimising selective pressures, becomes imperative to mitigate antimicrobial resistance.

## Abbreviations

ED: emergency department
GAS: group A Streptococcus
GP: general practice
HPSC: Health Protection Surveillance Centre (Ireland)
iGAS: invasive group A Streptococcus
IMSRL: Irish Meningococcal and Sepsis Reference Laboratory
